# Case of Traumatic Tetanus with Recovery

**Published:** 1888-11

**Authors:** Bernard Cohen

**Affiliations:** 228 Ellicott Street; Buffalo


### *CASE OF TRAUMATIC TETANUS WITH RECOVERY.*

By BERNARD COHEN, M. D., Buffalo.

Recoveries of traumatic tetanus are such rare occurrences that every case is worthy of report. To verify these premises, let me cite some of the statistics. G. A. B. McLeod, in his work on surgery of the war of Crimea, 1858, reports only two recoveries from twenty-three cases; he also reports one recovery in nineteen cases which occurred during the Indian war.

Alcock, an English surgeon, reports two recoveries in seventeen cases. Reference is made in the same work regarding the Egyptian campaign, where, of thirty cases, every one died within a week of the first appearance of the symptoms.

Hamilton, in his surgery, states that of 363 cases of traumatic tetanus, which occurred in our late war, only twenty-seven recovered; in his own practice he had thirteen, five of which recovered.

Jeremiah McCarty reports three cases with three deaths (*Heath's Dict. Surgery, p.* 599). The best results I came across were those of Dr. Frazer, an Edinburgh surgeon (*Amer. J. Med. Sciences*, Oct., 1868,); he had but two deaths among eleven cases.

Many experienced surgeons whom I interrogated could not recollect any recoveries in their practice. A friend of mine, who was house surgeon in Bellevue, said that of five cases occurring during his two years of service, not one recovered.

This array of statistics should be a sufficient excuse to publish, with some detail, a case which I had the opportunity of observing closely in the practice of my esteemed teacher, Dr. M. Hartwig, who kindly permits the publication.

Here, as in any case of recovery, the main interest centers in the treatment, which in this case was truly heroic. The maximum of medicine given in one day to this thirteen-year-old boy was as follows: Eserine, gr. 1½; coniine, gr. 1⅛; curare, gr. ⅙; bromide of potass. and bromide of soda, each, ʒ jss.; iodide of potass., gr. xvi.; antipyrine, gr. xxx.; chloral, ʒ v.; phenacetine, gr. xxii.; tinc. cannab. indicæ, ʒ i.; morphia sulph., gr. ½. Here I only counted the day as lasting twelve hours.

The whole duration of the tetanus, which followed twelve days after the injury, was forty days. The original cause was a wooden sliver which had pierced along under the skin of the middle finger. It was pulled out by a friend of the patient, after eight days. The first symptoms of the disease, consisting of stiffness of the jaws, with difficulty in swallowing, did not appear until four days after the sliver was pulled out. At that time, the wound was healed entirely. During the first two days the patient was merely observed, without treatment. The stiffness began spreading toward the back, and the exhibition of chloral and the bromides was begun. By-and-by the symptoms grew more intense, the continued stiffness being interrupted almost every minute with opisthotonos.

Consultations were held, and Drs. Lothrop, Fell and Tremaine saw the case in succession, and, with their consent, morphia, coniine, eserine and curare were successively added to the patient's diet. All of these remedies, however, were never fully sufficient to suppress the spells entirely. Even during his sleep I saw seizures of tetanic rigidity which lasted as long as ten minutes, the patient never waking up during that time.

One remarkable circumstance was that the arms never participated in a distinct manner, while in the legs the tetanus was noticed most of the time.

The urine was scanty, the temperature ran often to 103°, the bowels were frequently voided involuntarily, and at times diarrhœa was so persistent that it required the use of opium, tannin and bismuth to control it. The pulse remained usually in good order, but occasionally it ran to 120, and even to 160. At those times the dialysate of digitalis was used in five-drop doses, three times a day, with the expected improvement of the heart's action.

After the disease had lasted about two weeks in its full severity, free intervals between the tetanic spells began growing longer until, finally, the medicines were reduced to a quarter of the full doses, and then to naught.

As far as my personal observation during the administration of so many medicines could possibly determine, the subcutaneous injection of one-twelfth grain of curare (Merck's in water sol.) seemed not to have much of an effect, perhaps, though it lengthened the time between the spells when the disease was on its decline.

Eserine, one-twentieth of a grain subcutaneously, and internally in the same amount, seemed to have the best effect in allaying the intensity of the spasms, but it was probably the cause of the diarrhœa.

Chloroform did not at all please me in its action; it would only in a measure control the spasms, and it seemed as if the next one would come sooner and be longer in duration. It was, therefore, abandoned after a few trials.

The subcutaneous injections did not cause any abscesses to form, but one hard lump is still left from the use of the curare.

Take it all in all, it would be interesting to know the total quantity of medicines administered during those forty days. I am sorry that I cannot give the exact amounts, this owing to extraneous circumstances, but approximately the quantities were as follows: Chloral, ℥ij.; bromides, ℥iv.; eserine, 15gr.; coniine, 15 gr.; curare, ¾ gr.; morphia, 15 gr.; ex. opii, gr. iv.; dialysate of digitalis, ʒii.; antipyrine, ʒiv.; phenacetine, ʒi.; tinc. cannab. indicæ, ℥i. These quantities are truly astonishing, and certainly not overrated. They are worthy of notice to inspire others not to become homœopaths while treating this dreaded disease.

I could find but few cases of recovery from traumatic tetanus where the details of treatment were stated; and where such cases were recorded, the amounts of medicine sused were astonishing, for example: Dr. Lecato (*Med. News*, Nov., 1882, p. 593,) reports a case where the total amount of bromide of potash was half a pound, and two ounces of a strong tincture of calabar bean.

228 Ellicott Street.

